# SIRPα + CD209 + cell: a specialized antigen-presenting cell that contributes to anti-SIRPα/RT therapy in colorectal cancer

**DOI:** 10.1007/s00262-025-04025-z

**Published:** 2025-04-10

**Authors:** Yida Li, Weiqing Lu, Fan Xia, Yun Deng, Xin Jin, Yan Xuan, Yaqi Wang, Lijun Shen, Juefeng Wan, Hui Zhang, Yaqi Li, Xinxiang Li, Lili Huang, Zhen Zhang

**Affiliations:** 1 Department of Radiation Oncology, Fudan University Shanghai Cancer Center, Shanghai, 200032 China; 2 Department of Radiation Oncology, The Affiliated Suzhou Hospital of Nanjing Medical University, Suzhou Municipal Hospital, Gusu School, Nanjing Medical University, Suzhou, Jiangsu China; 3 Department of Oncology, Shanghai Medical College, Fudan University, Shanghai, 200032 China; 4 Department of Colorectal Surgery, Fudan University Shanghai Cancer Center, Shanghai, 200032 China

**Keywords:** Single-cell sequencing, SIRPα, CD209, Antigen-presenting cells, Radiation therapy, Colorectal cancer

## Abstract

**Objective:**

Colorectal cancer (CRC) is a leading cause of cancer-related mortality, with a need for improved treatment strategies. Antigen-presenting cells (APCs) have emerged as important modulators of immune responses in the tumor microenvironment (TME). This study aimed to explore the role of these cells in CRC and their potential synergy with radiation therapy (RT).

**Methods:**

Single-cell sequencing was performed before and after neoadjuvant therapy (NAT) to identify changes in myeloid cells within the tumor microenvironment, which was compared with peripheral blood of the same patients. The effect of RT with/without immunotherapy on these cells was evaluated in vivo and in vitro.

**Results:**

Single-cell sequencing showed that SIRPα + CD209 + cells are specialized antigen-presenting cells which are found to decrease in the TME while increasing in the peripheral blood after NAT. In vitro study confirmed their resistance to RT with further upregulated SIRPα expression and enhanced antigen presentation capability induced by RT. Moreover, these cells are involved in the superior tumor control by combination of RT and anti-SIRPα treatment.

**Conclusion:**

SIRPα + CD209 + APCs play a pivotal role in CRC immune modulation and show potential for synergy with RT. These cells could be a biomarker for antigen-presenting capacity, and enhancing their APC function could potentially improve RT/PD1 effectiveness by combination with anti-SIRPα in CRC.

**Supplementary Information:**

The online version contains supplementary material available at 10.1007/s00262-025-04025-z.

## Introduction

Colorectal cancer (CRC), the third most common malignancy worldwide, remains a major public health challenge due to its high mortality rate [1]. With the advent of immunotherapies, there is a growing need to discover new biomarkers and therapeutic strategies to overcome resistance, making it critical to unravel the complexities of the tumor microenvironment (TME). Macrophages, as key components of the mononuclear phagocyte system [2], play essential roles in maintaining immune homeostasis and demonstrate significant heterogeneity and multifunctionality in various diseases, including cancer [3–6]. The "don't eat me" signaling pathway, mediated by the SIRPα/CD47 axis, has gained substantial attention for its role in regulating macrophage phagocytosis and enabling tumor immune evasion [7].

Radiation therapy (RT), a localized treatment, not only damages tumor cells directly but also induces immunogenic cell death, thereby releasing tumor-associated antigens that stimulate antitumor immunity [8, 9]. The abscopal effect, which refers to an immune-mediated antitumor response occurring in non-irradiated regions, underscores the interplay between RT and the immune system [10]. More importantly, the synergistic effects of combining RT with immunostimulatory antibodies, such as anti-SIRPα agents, have shown promise in enhancing immunotherapy outcomes [11, 12]. Despite the potential of combination therapy, the most effective treatment regimens and timing remain under active investigation [13–16]. In the neoadjuvant setting, optimizing the synergy between RT and immunotherapy to modulate the TME and increase sensitivity to subsequent treatments is a key research focus.

This study employed single-cell sequencing to explore the changes in myeloid cells within the TME of patients with CRC undergoing neoadjuvant chemoradiotherapy. We specifically investigated the potential role of SIRPα + cells in antigen presentation and immune activation, as well as their response to combined RT and anti-SIRPα therapy. By identifying the dynamic shifts of these cells in the TME, we sought to provide a scientific basis for developing more effective immunotherapeutic strategies to improve outcomes in patients with CRC.

## Materials and methods

### Patient source

The 11 rectal cancer patient samples used for single-cell sequencing were collected from the clinical trial NCT03415763, which has been reviewed and approved by the ethics committee. The inclusion criteria for this study were: Lesions must be measurable by endoscopy, located within 12 cm of the anal verge, and histopathologically confirmed as rectal cancer; computed tomography, magnetic resonance imaging, and endoscopic ultrasonography (EUS) confirmed clinical stage III rectal cancer without organ involvement. Participants needed good organ function, no history of multiple primary cancers, and the ability to provide written informed consent. Exclusion criteria included: patients under 18 or over 75 years of age, a history of synchronous or metachronous malignancy within the last five years, intestinal obstruction, perforation, or bleeding requiring emergency surgery, previous pelvic irradiation, American Society of Anesthesiologists (ASA) physical status IV or V, pregnancy or breastfeeding, severe mental illness, and inability to tolerate surgery due to conditions like severe emphysema, interstitial pneumonia, or ischemic heart disease. Patients who cannot tolerate surgery; patients who have received steroid treatment within the last month; and patients who misunderstood the study’s conditions were also excluded. All included patients received neoadjuvant therapy (NAT) that included radiotherapy of 50 Gy/25F, along with concurrent capecitabine chemotherapy. Depending on the patients' disease remission or progression, the following interval chemotherapy treatments were administered: Two patients received the XELIRI regimen (Irinotecan combined with Capecitabine) once, 2 patients received the XELIRI regimen twice, 3 patients received the XELIRI regimen three times, and 1 patient received the FOLFOX regimen (Oxaliplatin, Fluorouracil combined with Leucovorin) four times during the interval period. Originally, paired specimens were planned for collection before and after neoadjuvant therapy, but pandemic restrictions in Shanghai caused some patients' surgeries to occur elsewhere, hindering specimen collection. Additionally, some patients achieved complete clinical remission after NAT, leading to observation and follow-up instead of surgery. Non-paired specimens were collected from other patients in the clinical trial who met the requirements post-NAT criteria. The clinical–pathological information was collected, as shown in Table 1.

**Table 1:** Characteristics of the 15 samples obtained from the 11 patients subjected to single-cell sequencing

Clinical–pathological characteristics	Pre-NAT	Post-NAT	Total
Gender			
Male	4	3	7
Female	4	4	8
Age, years (32–75, median 53)			
≤ 53	5	4	9
> 53	3	3	6
cT stage (baseline)			
T2	1	0	1
T3a	6	5	11
T3b	0	1	1
T4	1	1	2
cN stage (baseline)			
N0	1	1	2
N1	2	2	4
N2	4	4	8
Nx	1	0	1
Tumor regression grade			
TRG0	1	1	2
TRG2	2	4	6
TRG3	1	2	3
Unassessed	4	0	4
Microsatellite instability			
MSS	8	7	15

*NAT* neo-adjuvant therapy, *TRG* Tumor regression grade, *MSS* microsatellite stability

### Single-cell specimen acquisition

Biopsy specimens before NAT were collected by experienced clinicians. To avoid influencing postoperative pathological results, samples were taken 1 cm away from the ulcer formed by radiation, ensuring full thickness. Samples measuring 2–3 mm [3] were immediately immersed in pre-chilled single-cell preservation solution and transported at 4 °C as soon as possible for quality control and processing. RNA and peripheral blood mononuclear cells were also preserved for each sample.

### Single-cell analysis workflow

Single-cell suspensions were adjusted to 700–1,200 cells/μL. The 10 × Genomics Chromium Next GEM Single-Cell 5' Kit v2 (PN-1000263) was used following the User Guide. Cell suspension, master mix, gel beads, and oil were loaded into a chip to form microdroplets (GEMs), followed by reverse transcription and pre-amplification to generate full-length cDNA. The transcriptome sequencing library was constructed using a 10 × Genomics library construction kit (PN-1000190) and single-cell V(D)J Enrichment Kits [Human, TCR (PN-1000005)/BCR (PN-1000016)] and V(D)J Amplification Kits [Human, TCR (PN-1000252)/BCR (PN-1000253)]. Libraries were sequenced in high-throughput sequencing using the PE-150 mode at OE Biotech Co. Ltd (Shanghai, China).

### Cell culture

The cell lines used in this study included the THP-1 human monocytic cell line, the CT-26 murine CRC cell line, and the HCT-116 human CRC cell line, all of which were obtained from the Shanghai Institute of Cell Biology, Chinese Academy of Sciences. To induce differentiation into macrophages, THP-1 cells were seeded into six-well plates at a density of 1 × 10⁶ cells per well and induced to differentiate using 100 ng/mL Phorbol 12-Myristate 13-Acetate (PMA) per well. After 48 h, it was confirmed that the cells had attached and extended processes, after which the PMA-containing medium was removed by washing. For medium transfer experiments, HCT-116 tumor cells were cultured for 48 h, after which their supernatant was collected. This supernatant was then mixed with the complete culture medium of THP-1 cells in a 1:1 ratio to serve as the conditioned medium for the THP-1 cells. The medium for the THP-1 cells was changed every 2 days, with each change involving a 1:1 addition of the supernatant from HCT-116 cultures, until the culture period reached 96 h.

### Animals

Forty 8-week-old, male BALB/c mice were purchased from Beijing Vital River Laboratory Animal Technology Co., Ltd. The mice were housed five per cage under standard laboratory conditions (22 ± 2 °C, 55 ± 10% humidity, and a 12-h light–dark cycle). All animal experimental procedures were conducted in accordance with the guidelines of the Institutional Animal Care and Use Committee of Fudan University and were approved by the Department of Experimental Animal Science (approval number 202011014S). After 1 week of acclimation, each mouse was inoculated with 5 × 10 [5] CT-26 cells in the left flank. Eight days later, the mice were marked with ear tags for individual identification, and 32 mice were randomly divided into eight groups of four each. Day 1 of the first RT or immunotherapy session was designated as D1. The eight groups were as follows: (1) Control group: no irradiation and no immunotherapy. (2) RT group: received 8 Gy irradiation on D1 and D3, but no immunotherapy. (3) RT + anti-PD-1 group: received 8 Gy irradiation on D1 and D3, followed by intratumoral injection of 200 μg of PD-1 antibody on D4, D7, and D10. (4) RT + anti-SIRPα group: received 8 Gy irradiation on D1 and D3, followed by intratumoral injection of 200 μg of SIRPα antibody on D4, D7, and D10. (5) RT + dual-drug group: received 8 Gy irradiation on D1 and D3, followed by intratumoral injection of both 200 μg of PD-1 and 200 μg of SIRPα antibodies on D4, D7, and D10. (6) Anti-PD-1 group: received intratumoral injection of 200 μg of PD-1 antibody on D1, D4, and D7. (7) Anti-SIRPα group: received intratumoral injection of 200 μg of SIRPα antibody on D1, D4, and D7. (8) Dual-drug group: received intratumoral injection of both 200 μg of PD-1 and 200 μg of SIRPα antibodies on D1, D4, and D7. Mice that did not require irradiation underwent sham irradiation, while those that did not receive immunotherapy were injected intratumorally with an equivalent volume of saline. Tumor long and short diameters were measured on even-numbered days starting from D0. The tumor surface area was calculated by multiplying the long diameter by the short diameter. On D12, the mice that were required to be killed were euthanized by dislocation of the cervical vertebrae, and specimens were collected.

### Radiation

Radiation for the mice was administered by the Small Animal Radiation Research Platform (SARRP) at the Fudan University Cancer Center, China. All mice were anesthetized using isoflurane for induction and maintenance (induction increasing from 0.5 to 2.0% over 8 min, maintenance at 1.5%, adjusted according to the animal's condition) and then immobilized using a custom-designed box that restrained their limbs and stretched their bodies. A 2.5-cm-thick lead shield was used to cover all areas except the region near the tumor to protect other parts of the body from radiation. Subsequently, each mouse was irradiated with a dose of 8 Gy using a 6-MV linear accelerator (Siemens Primus-Hi). For the control group, each mouse received 0 Gy of irradiation. The dose rate was 2 Gy/min. The source-to-surface distance was set at 1 m, and the size of the radiation field was 2.5 × 15 cm.

### Flow cytometry

To prepare a single-cell suspension, first, the tissue samples were minced into small pieces. Then, they were treated with a solution of RPMI-1640 (Gibco Inc.) containing Type IV collagenase (1 mg/ml) and DNase I solution (0.01 mg/ml; Sigma-Aldrich) using Gentle MACS (Miltenyi Biotec) for 30 min. The resulting suspension was passed through a 40-μm pore size filter. Next, red blood cells were removed by incubating the sample with red blood cell lysis buffer (10 × ; Sigma-Aldrich) at the required concentration in the dark for 15 min. Finally, a dead cell removal kit (Miltenyi Biotec) was used to ensure that cell viability was greater than 90%.

Well-prepared single-cell suspensions were centrifuged and stained with live/dead dye and incubated on ice in the dark for 20 min. After neutralization, the cells were resuspended in stain buffer containing 1 μL Human TruStain FcXTM (Biolegend, catalog number 422302), incubated on ice for 10 min, and centrifuged again. Surface staining suspensions were prepared using APC anti-SIRPα (Biolegend, clone SE5A5), PE anti-CD209 (Biolegend, clone 9E9A8), PECy7 anti-HLA-DR (Biolegend, clone L243), and APCCy7 anti-CD11b (Biolegend, clone M1/70) at concentrations provided by the manufacturer. Gating strategies are shown in Supplementary Fig. S1. All flow cytometry stainings included single-antibody controls and fluorescence minus one (FMO) controls. Data were acquired on a BD Biosciences LSRII FORTESSA flow cytometer and analyzed using FlowJo software (v9.3.2, Tree Star).

### Statistical analysis

For this study, high and low thresholds for various parameters were defined as the median. The SPSS software (version 17.0) was used to build databases and conduct statistical analyses. For comparisons between two groups, if the normal distribution and homogeneity of variance were satisfied, a t test was used; otherwise, the Wilcoxon rank-sum test was used. Graphical representation of statistical analysis was carried out using SPSS, GraphPad Prism version 6.0, and R software package version 4.0.3. All statistical significance was two-sided, and p < 0.05 was considered statistically significant.

## Results

### Depletion of SIRPα + myeloid cells after NAT

**Fig. 1:**
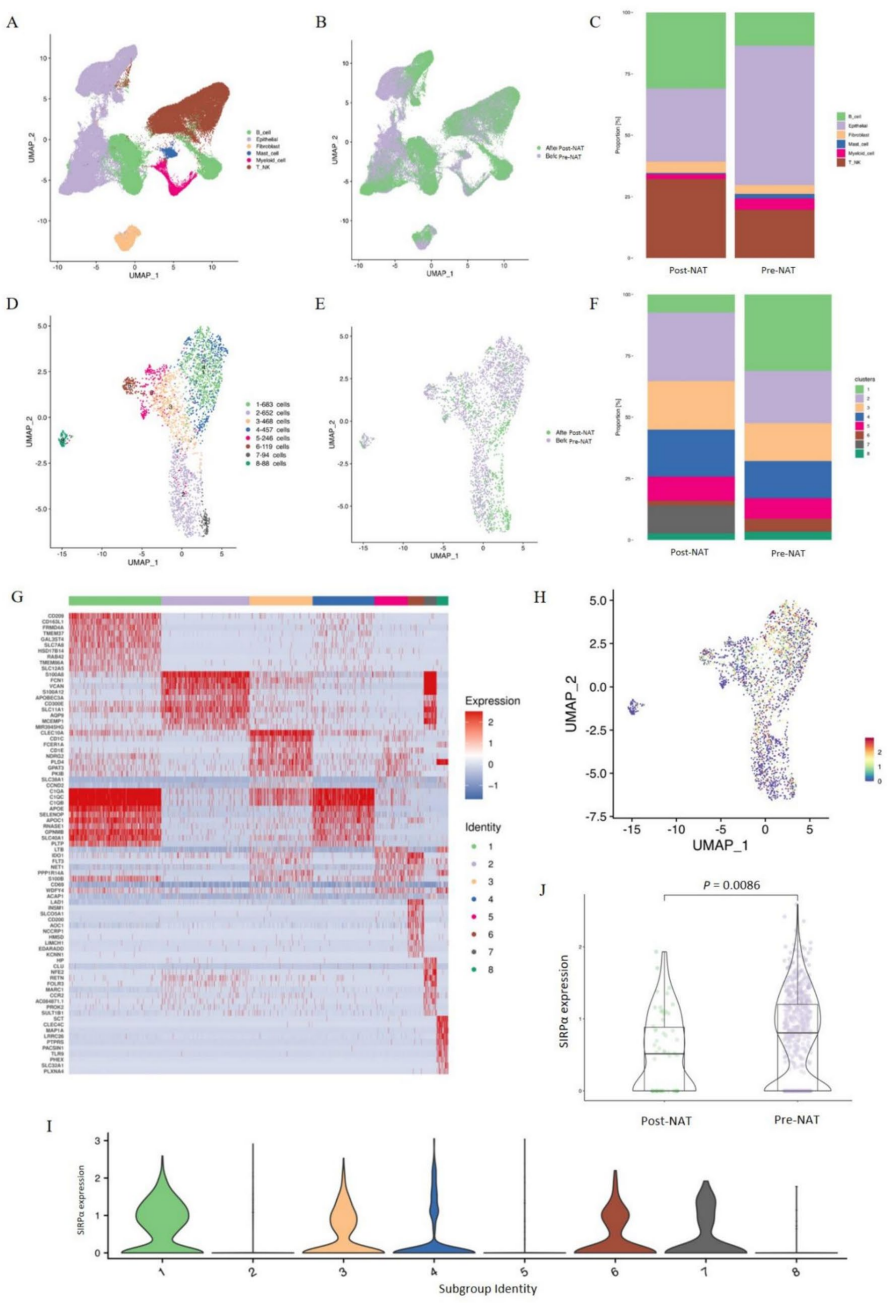
Single-cell sequencing revealed SIRPα + myeloid cells. **A** UMAP plot showing cell type annotations. **B** Distribution of cell subpopulations before and after neoadjuvant chemoradiotherapy (NAT). **C** Changes in the proportion of each cell type pre- and post-NAT. **D** Unsupervised dimensionality reduction clustering results of myeloid cells. **E** Distribution of myeloid cell subpopulations before and after NAT. **F** Proportional changes in the proportion of each myeloid cell subtype pre- and post-NAT. **G** Marker genes for each myeloid cell subpopulation, with red indicating high expression and blue indicating low expression. **H** Single-gene mapping of *SIRPα* in myeloid cells, with color representing *SIRPα* abundance. **I** Distribution of SIRPα + cells across different myeloid cell subpopulations. **J** Comparison of *SIRPα* gene expression between the post- and pre-NAT groups

Using single-cell resolution techniques, we performed quality control and analyzed 62,116 cells from pre-RT samples and 48,345 cells from post-RT samples (see Supplementary Fig. S2). From this analysis, we identified myeloid cells (Fig. 1A). Upon comparing the pre- and post-NAT samples, we observed a significant reduction in both the total number and proportion of myeloid cells post-NAT. In contrast, the proportions of B cells, fibroblasts, T cells, and natural killer (NK) cells increased following NAT (Fig. 1B, C).

Given recent advances in our understanding of SIRPα + macrophages, we focused on exploring the role of SIRPα + antigen-presenting cells (APCs) in CRC. To do so, we conducted a second round of unsupervised clustering analysis on myeloid cells. This analysis revealed the presence of eight distinct subpopulations (Sub), with Sub1 and Sub2 being the most prominent (Fig. 1D). By comparing pre- and post-NAT samples, we discovered that the reduction in Sub1 cells was the most pronounced post-NAT (Fig. 1E, F). We then proceeded to identify signature genes for each subpopulation (Fig. 1G). *CD209* emerged as the most significant signature gene for Sub1, whereas *S100A8* was the standout signature gene for Sub2. In summary, the total number of myeloid cells declined post-NAT, with this decline largely attributable to a significant reduction in the CD209 + subpopulation.

In a subsequent analysis, we examined the expression profile of the SIRPα gene across all identified subpopulations (Fig. 1H, I). We observed that *SIRPα* was notably enriched in Sub1. Given the substantial decrease in the overall number of myeloid cells post-NAT, the percentage of *SIRPα*-expressing cells declined from 43% pre-NAT (mean 21%, SD 0.11) to 24% post-NAT (mean 37.2%, SD 0.14). The absolute number of SIRPα + myeloid cells dropped to less than one-quarter of the pre-NAT levels. When comparing *SIRPα* expression levels pre- and post-NAT within each subpopulation, we identified a significant reduction in *SIRPα* gene expression across all clusters (Fig. 1J). Overall, our findings indicate a pronounced downregulation of *SIRPα* gene expression post-NAT, primarily within the CD209 + cluster, and a concurrent decrease in the population of SIRPα + CD209 + double-positive cells following RT.

### CD209 + SIRPα + cells exhibit specialized antigen-presenting functions

**Fig. 2:**
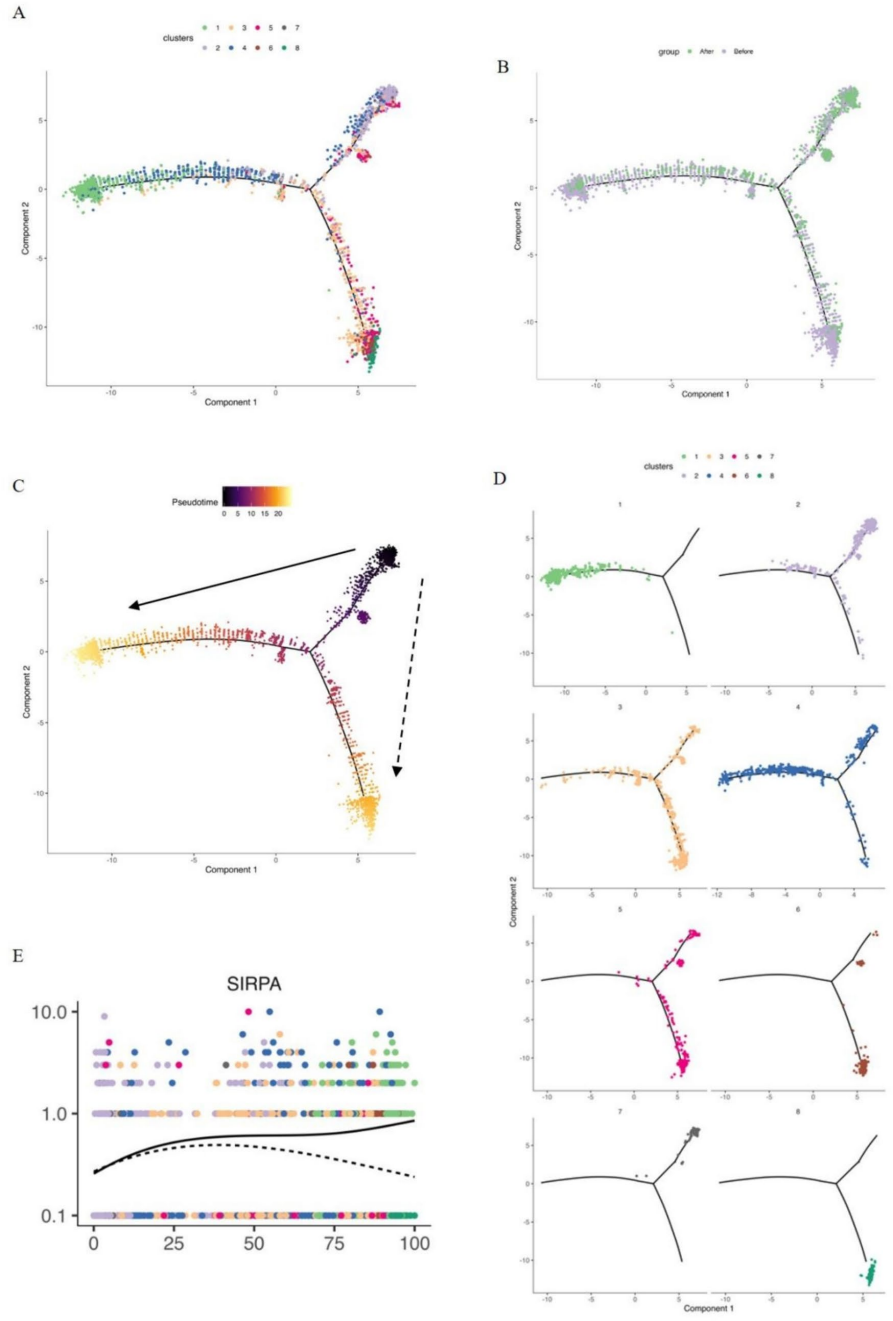
Pseudotime analysis based on the single-cell sequencing. **A** Pseudotime trajectory of myeloid cell differentiation for each cell cluster, with colors representing different cell clusters and branches indicating potential decision points in biological processes. **B** Distribution of cells from pre- and post-neoadjuvant therapy (NAT) samples along the pseudotime axis. **C** Pseudotime trajectory of myeloid cells, with colors transitioning from dark to light to indicate the progression of differentiation from early to late stages. **D** Mapping of each cell cluster onto the differentiation trajectory, showing differentiation relationships. **E** Changes in *SIRPα* gene expression along the pseudotime in myeloid cells, with the vertical axis showing *SIRPα* expression levels, the horizontal axis showing pseudotime, and solid and dashed lines representing *SIRPα* expression along the trajectories State1-2-7 and State3, respectively

To better understand the developmental states of CD209 + SIRPα + cells in relation to other myeloid cells, we performed pseudotime analysis on 2,807 myeloid cells to construct a developmental trajectory (Fig. 2A, B). By mapping the distribution of signature genes for each myeloid subpopulation along this trajectory, we identified the starting and end points (Fig. 2C). Sub2 was defined as the starting point due to its monocyte-like characteristics, whereas Sub1 was a terminal point, with its signature genes suggesting a trajectory toward macrophage differentiation (Fig. 2D). *SIRPα* expression progressively increased along the pseudotime axis, but following a bifurcation point, the expression of *SIRPα* began to decline (Fig. 2E). This pattern highlighted the maturation and specialization of SIRPα + CD209 + cells within the broader developmental landscape of myeloid cells, indicating that these cells likely possess distinct functional properties.

**Fig. 3:**
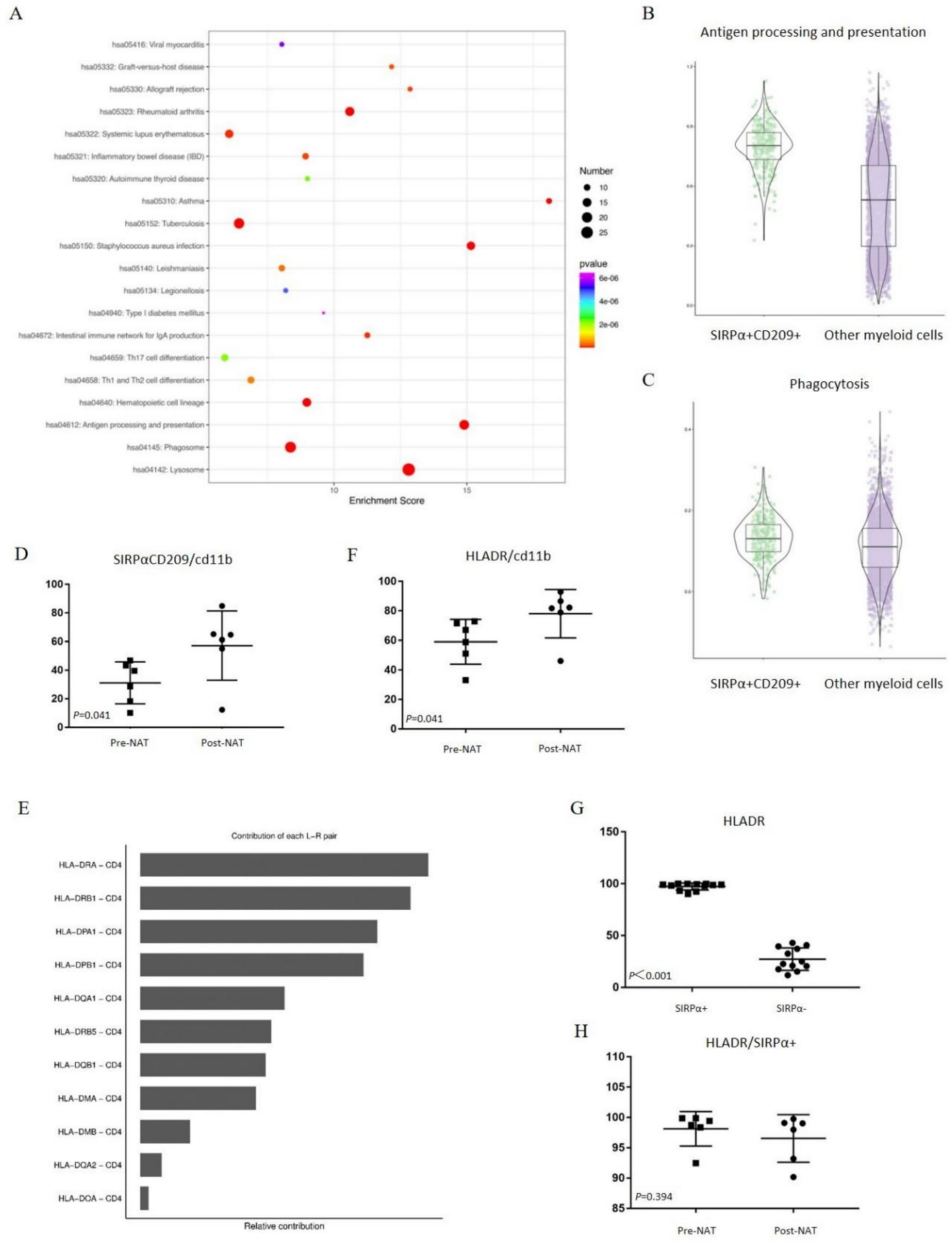
SIRPα + CD209 + cells in peripheral blood exhibit enhanced antigen presentation function. **A** KEGG enrichment analysis of DEGs between CD209 + SIRPα + double-positive cells and other myeloid cells. Bubble size represents the number of genes in the intersection between the genes in the pathway and the input gene list. The color of the bubbles indicates the P-value. **B** Antigen processing and presentation functional gene signature scores for SIRPα + CD209 + cells versus other myeloid cells. **C** Phagocytosis functional gene signature scores for SIRPα + CD209 + cells compared to other myeloid cells. **D** Proportion of SIRPα + CD209 + cells among CD11b + cells in peripheral blood mononuclear cells (PBMCs) before and after neoadjuvant therapy (NAT). **E** Relative contribution of each receptor–ligand interaction within MHCII class molecules to the overall interaction strength. **F** Ratio of HLA-DR + cells to CD11b + cells in peripheral blood before and after NAT. (G) Comparison of *HLA-DR* expression in all CD11b + cells before and after NAT and between SIRPα + cells and SIRPα- cells. **H** Ratio of HLA-DR + cells to SIRPα + cells in peripheral blood before and after NAT

To further explore the specialized functions of SIRPα + CD209 + cells, we conducted GO and KEGG enrichment analyses on DEGs between SIRPα + CD209 + cells and other myeloid cell populations. These analyses revealed that SIRPα + CD209 + cells were enriched in pathways associated with the immune response, antigen processing and presentation, as well as major histocompatibility complex II (MHCII) class molecule-related pathways (Supplementary Fig. S3, Fig. 3A).

Considering the well-established role of SIRPα in regulating phagocytic functions [17], we performed gene set scoring on SIRPα + CD209 + cells and other myeloid cells, focusing on gene sets related to antigen processing and presentation, as well as phagocytosis. As shown in Fig. 3B, SIRPα + CD209 + cells displayed significantly enhanced antigen processing and presentation functions (p < 0.001, median 0.80 vs. 0.53) compared to other myeloid cells. However, while phagocytic function also showed a statistically significant difference, it was not as biologically meaningful (Fig. 3C, P < 0.001, median 0.13 vs. 0.11).

### Increased SIRPα + CD209 + cells in peripheral blood post-NAT with upregulated antigen presentation

We analyzed the changes in circulating CD209 + SIRPα + double-positive cells pre- and post-NAT using peripheral blood samples taken concurrently with the single-cell specimens. As shown in Fig. 3D, the proportion of CD209 + SIRPα + double-positive cells within the CD11b + myeloid population significantly increased following neoadjuvant chemoradiotherapy (P = 0.041). This finding contrasts sharply with the tissue-based single-cell sequencing results, which showed a decline in CD209 + SIRPα + cells within the TME. This discrepancy underscores the importance of further research into the migration patterns and dynamic behavior of these cells post-irradiation.

To further assess the antigen presentation functionality, we analyzed h*uman leukocyte antigen DR* (*HLA-DR)*, a key MHCII molecule, as a surface marker for APCs. *HLA-DR* expression was tested in the same peripheral blood samples (Fig. 3E). The results, depicted in Fig. 3F, showed a significant upregulation of *HLA-DR* expression in CD11b + cells post-NAT (P = 0.041), reflecting an overall enhancement in the antigen presentation capacity of circulating APCs. Interestingly, this increased antigen presentation ability was primarily driven by the rising proportion of SIRPα + cells in the peripheral blood. Specifically, *HLA-DR* expression in this SIRPα + macrophage population was significantly higher in SIRPα + macrophages compared to SIRPα-negative macrophages (Fig. 3G, P < 0.001). Furthermore, while the overall antigen presentation function of circulating macrophages changed before and after NAT, the capacity of these SIRPα + macrophages to present antigens remained consistently elevated (Fig. 3H).

### SIRPα + CD209 + cells display radioresistance and increased antigen presentation post-RT

**Fig. 4:**
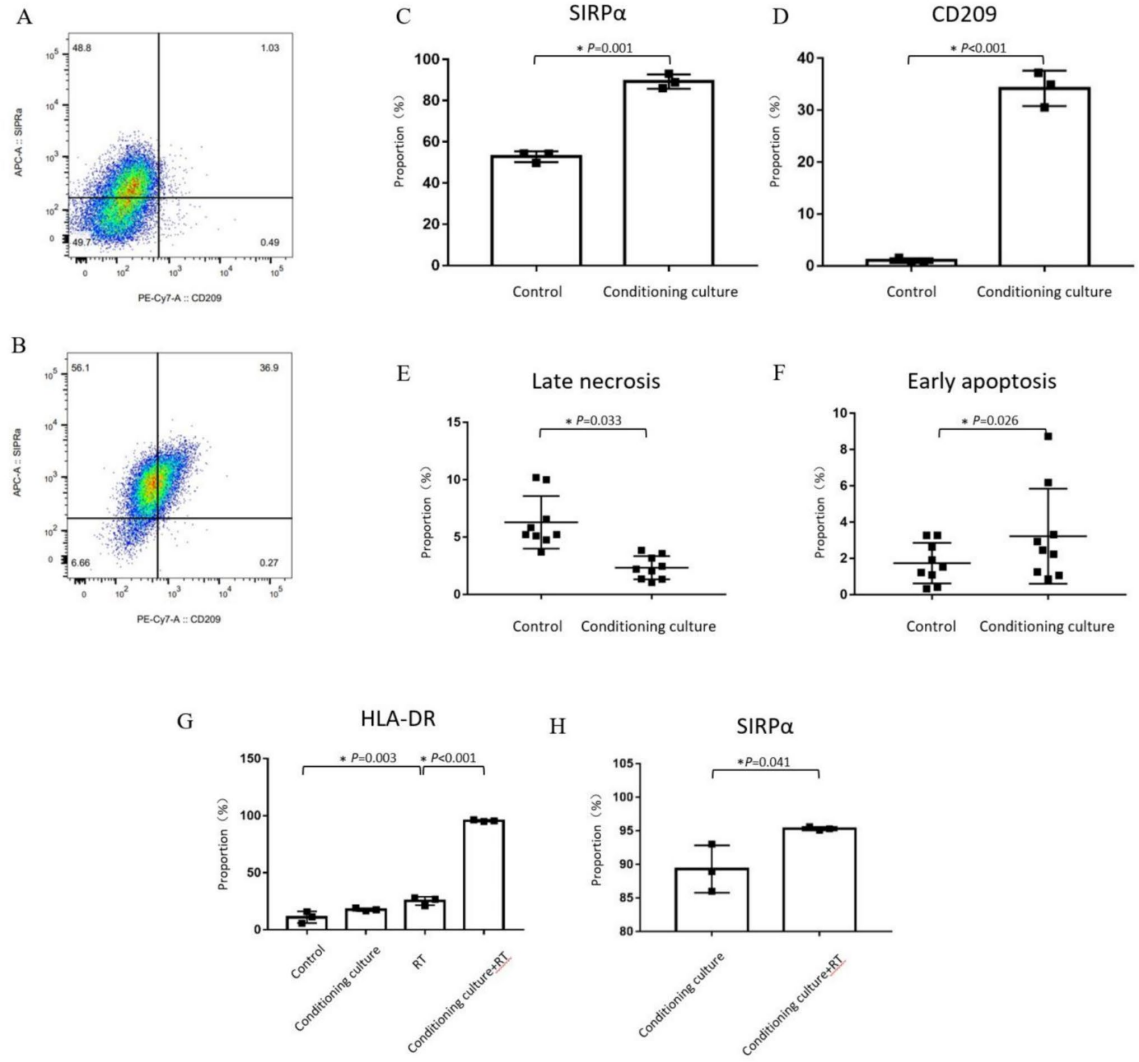
Regulation of SIRPα + CD209 + cells by radiation in vitro. **A** Flow cytometry plots showing *SIRPα* and *CD209* expression in THP-1-derived macrophages. **B** Flow cytometry plots of *SIRPα* and *CD209* expression in macrophages after 96 h of conditioning culture. **C** Comparison of *SIRPα* expression and **D** *CD209* expression in macrophages after 96 h of conditioning culture. **E** Percentage of late necrotic cells and **F** percentage of early apoptotic cells in THP-1-derived macrophages, either conditioning cultured or not, and irradiated with 5 Gy or not. **G** Differences in *HLA-DR* expression in macrophages with or without conditioning culture and irradiation. **H** Comparison of *SIRPα* expression in conditioning cultured macrophages that were irradiated with 5 Gy versus non-irradiated controls. Bar charts show the means and standard deviations, with stars indicating significant differences

After successfully inducing SIRPα + CD209 + double-positive cells via medium transfer experiment (Fig. 4A-D), we investigated differences in radiosensitivity compared to uninduced macrophages and examined changes in antigen presentation function post-RT. Macrophages generated were exposed to 5 Gy of radiation, and 24 h later, both adherent cells and those in the supernatant were collected for analysis. The results revealed a significant decrease in the proportion of late necrosis (Fig. 4E, P = 0.033) and increase in early apoptosis of SIRPα + CD209 + cells (Fig. 4F, P = 0.026 representative images are shown in Supplementary Fig. S1B). These findings suggest that SIRPα + CD209 + cells exhibit a notable resistance to radiation.

We further assessed changes in antigen presentation function by comparing *HLA-DR* expression in macrophages with or without medium transfer and radiation exposure (Fig. 4G). Both radiation (P = 0.003) and medium transfer (P = 0.125) contributed to different levels of *HLA-DR* upregulation in THP-1-derived macrophages. However, the most significant increase in *HLA-DR* expression occurred when cells were conditioning cultured and exposed to radiation (P < 0.001) compared to the irradiation group alone.

To further explore the impact of radiation on *SIRPα* expression, we exposed conditioning cultured macrophages to 5 Gy of radiation and compared *SIRPα* expression in irradiated versus non-irradiated cells (Fig. 4H). Radiation exposure significantly increased *SIRPα* expression in SIRPα + CD209 + cells induced by conditioning culture (P = 0.041).

These in vitro findings demonstrate that SIRPα + CD209 + cells are radioresistant and exhibit significant upregulation of *HLA-DR*, independent of the presence of tumor cells, following radiation exposure. Simultaneously, radiation also enhances *SIRPα* expression, further supporting the radioresistance and specialized antigen presentation capabilities of these cells.

### Potential synergistic mechanism between anti-SIRPα treatment and RT

**Fig. 5:**
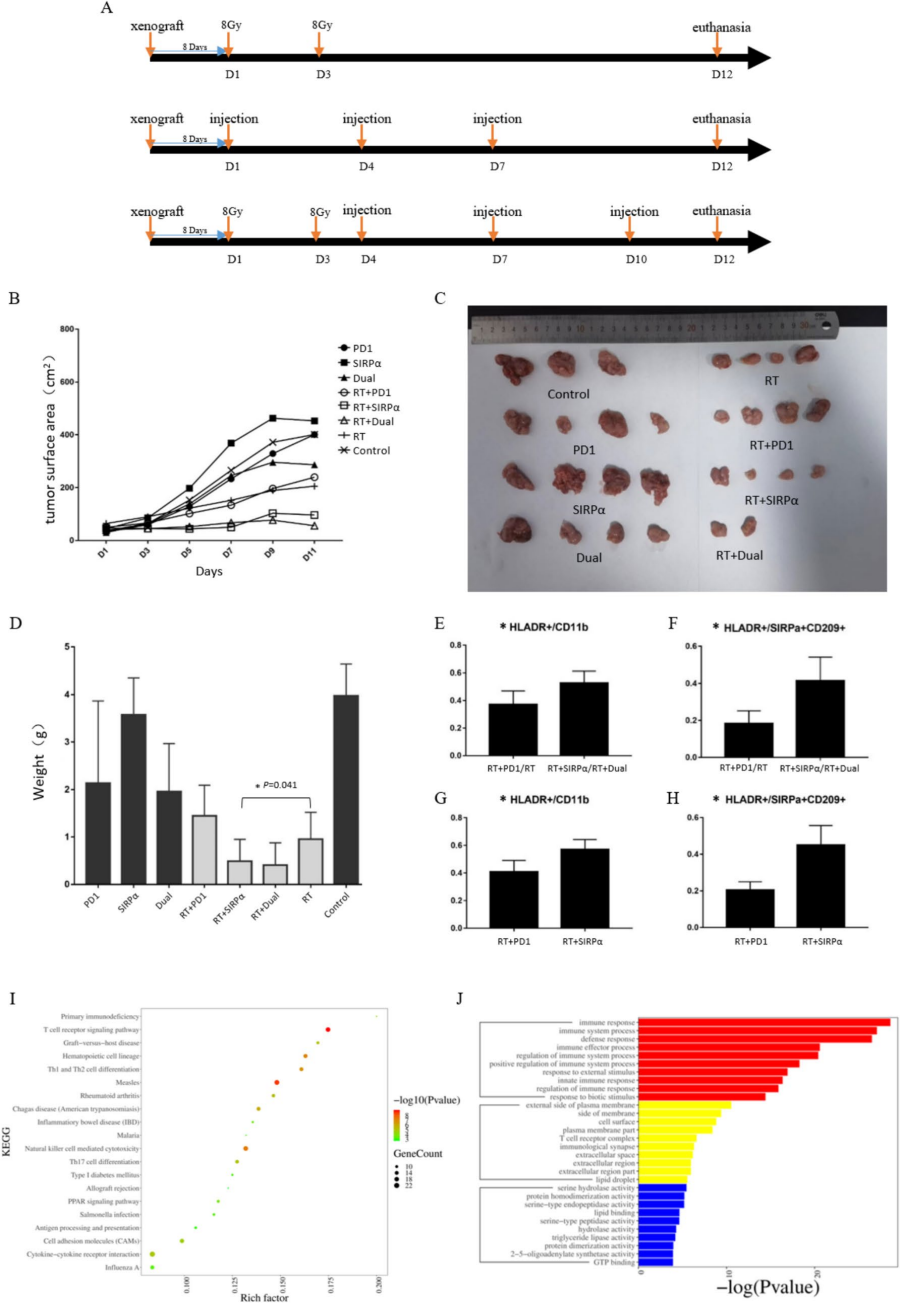
Role of SIRPα + CD209 + cells in the combination of anti-SIRPα and radiation therapy (RT) in vivo. **A** Intervention time points for the groups receiving RT alone, immunotherapy alone, and the combination of RT and immunotherapy. **B** Changes in tumor surface area over time following the start of the intervention. **C** Tumor grouping after excision, noting that one animal in the control group died due to non-tumor-related reasons, while two animals in the radiation plus dual-drug group achieved complete remission. **D** Comparison of tumor weights among the groups. **E**–**H** Flow cytometry results for various cell types and their proportions in groups treated with RT combined with SIRPα and/or PD1. Bar charts present the means and standard deviations, with stars indicating significant differences. **I** The KEGG and **J** GO analyses of the transcriptome sequencing results for differentially expressed genes between the RT plus SIRPα group and the RT plus PD1 group

Using eight groups of mouse xenograft models, we established PD1, SIRPα, dual-drug, radiation + PD1, radiation + SIRPα, radiation + dual-drug, radiation, and control groups, with specific intervention time points as shown in Fig. 5A. Tumor growth was monitored every other day starting from Day 1 by measuring both the long and short diameters of the tumors to roughly estimate the tumor area (Fig. 5B). SIRPα monotherapy showed no benefit, while dual-drug treatment was superior to PD1 monotherapy. All radiation groups had markedly slower tumor growth compared to non-radiation groups, with radiation + SIRPα showing greater synergy than radiation + PD1. Furthermore, the best efficacy was observed in the radiation + dual-drug group. Figure 5C displays the dissected tumors. One mouse in the control group died during the experiment for non-tumor reasons, and two mice in the radiation + dual-drug group achieved complete remission, resulting in no tumor tissue. We weighed each tumor, as shown in Fig. 5D; the tumor weight in the radiation + dual-drug group was significantly lower than in the radiation + PD1 group (P = 0.041), validating the effectiveness of combining anti-SIRPα with RT to enhance the anti-tumor effects.

To elucidate the mechanism behind the superior tumor control observed in the RT and SIRPα blockade combination, we used flow cytometry to measure *SIRPα*, *CD209*, and *HLA-DR* expression in tumor tissues from the xenograft models. We specifically investigated the synergistic mechanism of RT and SIRPα blockade by comparing radiation groups without anti-SIRPα (radiation + PD1, radiation alone) and those with anti-SIRPα (radiation + SIRPα, radiation + dual-drug). As demonstrated in Fig. 5E and F, combining radiation with anti-SIRPα significantly upregulated *HLA-DR* expression in CD11b + cells (P = 0.004), with an even greater effect observed in SIRPα + CD209 + cells (P < 0.001). Similar results were obtained when comparing the radiation + SIRPα group to the radiation + PD1 group (Fig. 5G and H, P = 0.029 and P = 0.005, respectively).

To further investigate the underlying mechanisms of this synergy, we performed transcriptome sequencing on tumor tissues from each group. GO and KEGG enrichment analyses were conducted on the DEGs to explore the distinct effects of combining RT with SIRPα antibody versus RT with PD1 antibody. The comparison revealed that the DEGs were enriched in immune response functions (Fig. 5I) and in Th cell and TCR-related pathways (Fig. 5J). This suggests alterations in T-cell function. Additionally, the Reactome pathway enrichment analysis (https://reactome.org/) produced similar results, emphasizing the promotion of TCR signaling pathways, particularly ZAP-70 and TCR signal transduction (Supplementary Fig. S4).

## Discussion

In this study, we investigated the expression characteristics of *SIRPα* in myeloid cells within the CRC microenvironment. Previous single-cell studies of CRC have primarily identified two primary myeloid cell clusters: C1QC + and SPP1 +  [18, 19]. However, in our study, neither pre- nor post-NAT samples revealed a distinct cell cluster with high *SPP1* expression. This discrepancy may arise from our inclusion of post-NAT samples, whereas earlier studies predominantly analyzed treatment-naive samples. Additionally, all of the patients in our study had rectal cancer rather than colon cancer, suggesting potential differences in the TMEs of these cancers, as supported by variations in the T-cell profiles [20].

To the best of our knowledge, SIRPα + CD209 + cells have only been previously mentioned once [21]. That study subclustered HLADR + SIRPα + cells and identified a subset characterized by CD209/MERTK/MRC1/CD163L1, referred to by the authors as CD163hi macrophage-like cells, focusing on their antigen presentation interactions with Th cells. This closely parallels our findings of SIRPα + CD209 + cells and their location in Sub1. Although SIRPα is commonly linked to phagocytic function, our study suggests it may also influence antigen presentation, which diverges from conventional views. It is possible that *SIRPα* upregulation in APCs could serve as a mechanism to halt phagocytosis and enhance antigen presentation, but this hypothesis requires further validation. Other studies have also noted similar synergies between SIRPα and HLA-DR, such as the report of a SIRPα + HLADR + CD1c + subset as mature dendritic cells in the lymph nodes [21] and CD14 + CD64 + HLA-DR + SIRPα + monocytic phagocytes involved in CD4 + T-cell activation [22]. Collectively, these studies, including ours, suggest a potential role for SIRPα in enhancing the antigen presentation function of APCs.

A distinctive aspect of our study is the dual perspective of pre- and post-NAT analysis from two perspectives: before and after NAT, and the comparison between local tissue and peripheral blood. We observed a decrease in local mature myeloid cells and an increase in monocyte infiltration post-NAT, consistent with other studies reporting RT-induced clearance of some mature, suppressive phenotype macrophages [23, 24]. The promotion of monocyte infiltration by RT aligns with findings in other tumor types [25].

Traditional views suggest that APCs mature locally and migrate to lymph nodes [26–28]; however, additional evidence and a more detailed mechanistic understanding are required [29]. Our findings indicate a significant decrease in tissue myeloid cells, including SIRPα + CD209 + cells, post-RT. Three potential reasons for this reduction are: (1) Cell migration, as evidenced by the increased SIRPα + CD209 + cells in peripheral blood post-RT, which is consistent with findings in other tumor studies [30]. (2) Cell death, while compared to typical macrophages, the radiosensitivity of SIRPα + CD209 + cells is lower. (3) Phenotypic change, supported by our finding and others [31] that RT can upregulate *SIRPα*. We propose that during NAT, the massive antigen release leads to antigen uptake by APCs, which then upregulate *SIRPα* to facilitate antigen presentation and migration to the peripheral blood and circulation to the lymph nodes to exert antigen presentation functions. This study provides concrete cellular insights into the mechanisms underlying APC functionality, possibly serving as a basis for understanding the abscopal effect of RT, a concept supported by another study reporting that the abscopal effect of RT combined with CD47 antibodies depends on macrophage migration [32].

Regarding the combination of RT and anti-SIRPα + in animal experiments, previous studies [33] reported that the combination of RT, anti-SIRPα, and anti-PD-1 reverses immune tolerance and enhances T-cell activation through improved antigen presentation. Enhanced dendritic cell function was cited as a key factor in improved CD8 + T-cell activation. Similarly, another study [31] reported that RT combined with anti-SIRPα or concurrently with anti-PD1 showed antitumor effects. Unlike these studies focusing on tumor control, our research focused on exploring the specific impact of RT and antibodies on antigen presentation, particularly why the combination of RT and anti-SIRPα outperformed anti-PD1. We found that the synergy of RT and anti-SIRPα indeed enhances antigen presentation function and T-cell activation, likely through the activation of SIRPα + CD209 + cells. Our study sheds light on the potential role of specific cellular phenotypes in the intermediate steps bridging RT and immunotherapy to T-cell activation through enhanced antigen presentation. Pre-treatment detection of SIRPα + CD209 + cells or *SIRPα* expression in tumors could serve as significant biomarkers for the application of RT and SIRPα antibody therapy. Patients with high levels of SIRPα + CD209 + cells or *SIRPα* expression in tumors may benefit from this approach, especially if resistant to PD1 therapy.

A limitation of this study is its preclinical nature. Future studies are needed to validate these findings in clinical settings. The second limitation lies in our animal experiments, including the setup of control groups and the number of animals. However, given that our primary objective was to obtain samples rather than compare therapeutic effects, and considering that we avoided direct comparisons between single-antibody treatments and control groups during subsequent analyses, we have endeavored to minimize issues arising from the design of our animal experiments. Additionally, our research focusing on single-cell group lacks an exploration of its interactions with other related cells, which limits further understanding of this cell type. Further validation through functional cellular experiments is planned, including spatial and temporal studies of tumor-associated macrophages through tracing techniques after irradiation and further examination of the antigen-presenting functions of SIRPα + macrophages in different types of cancers.

In conclusion, our single-cell sequencing data of myeloid cells before and after NAT identified a specialized antigen-presenting SIRPα + CD209 + cell subset. These cells decreased in the TME but increased in peripheral blood post-NAT, exhibiting RT resistance, and upregulated antigen presentation capacity after RT. The combination of RT and anti-SIRPα showed superior tumor control compared to anti-PD1, which is potentially due to the enhanced antigen presentation capacity of SIRPα + CD209 + cells. SIRPα + CD209 + cells could be a biomarker for antigen-presenting capacity, and enhancing their APC function could potentially improve RT/PD1 effectiveness by combination with anti- SIRPα in CRC.

## Supplementary Information

Below is the link to the electronic supplementary material.

Supplementary file 1 (PDF 425 KB)

## Data Availability

Sequence data that support the findings of this study are stored in an institutional repository and will be shared upon request to the corresponding author.
