# Supplementary material for: SIRPα + CD209 + cell: a specialized antigen-presenting cell that contributes to anti-SIRPα/RT therapy in colorectal cancer
Source: Cancer Immunol Immunother. 2025 Apr 10;74(5):167. doi: 10.1007/s00262-025-04025-z (PMC11985876; doi:10.1007/s00262-025-04025-z)
Supplement: Supplementary file 1 — (PDF 425 KB) [file 262_2025_4025_MOESM1_ESM.pdf]

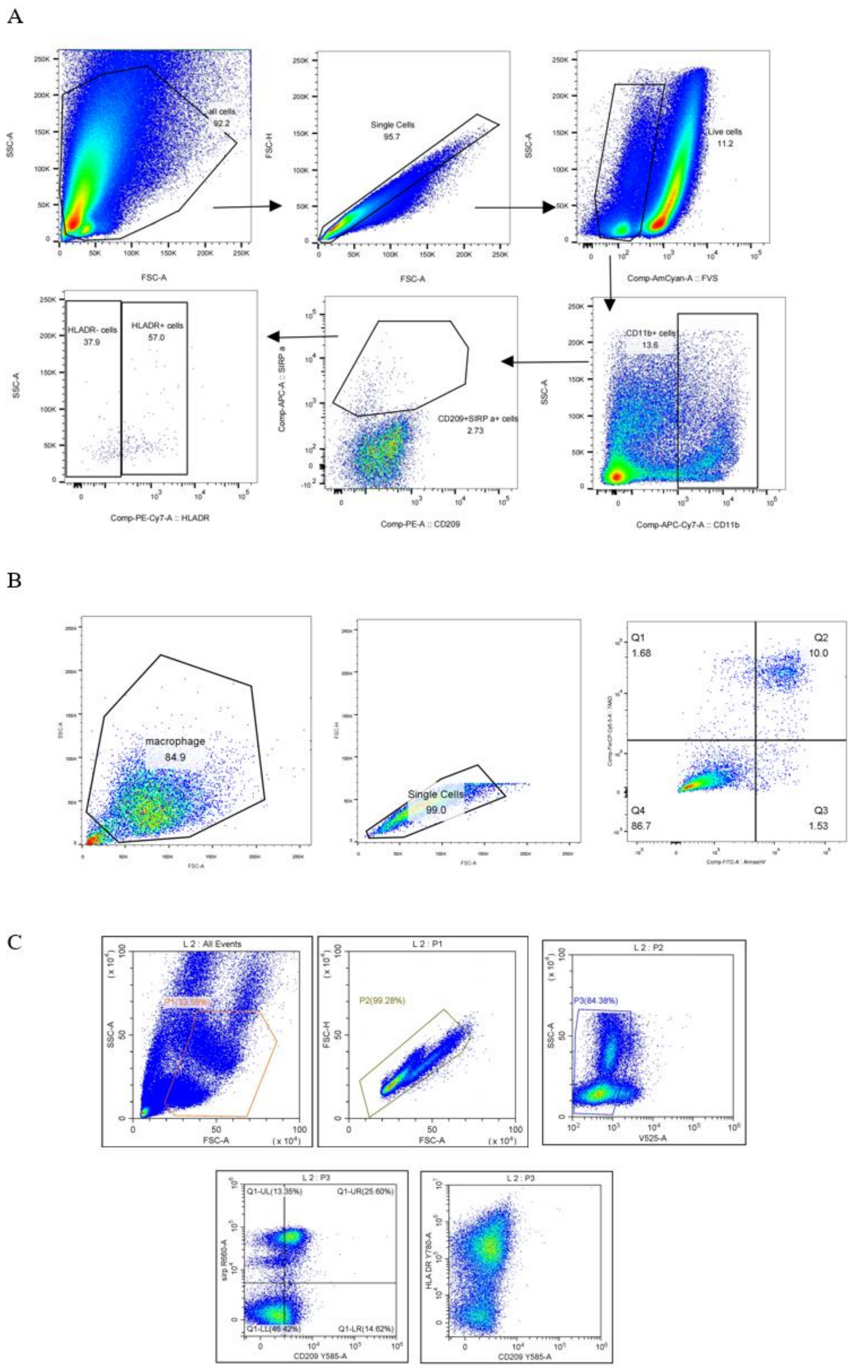

Supplementary Fig. S1. Gating strategies showing: (A) for Fig. 3 D, F, G, H and Fig. 5 E, F; (B) for Fig. 4 E, F, G, H; (C) for Fig.4 G, H.

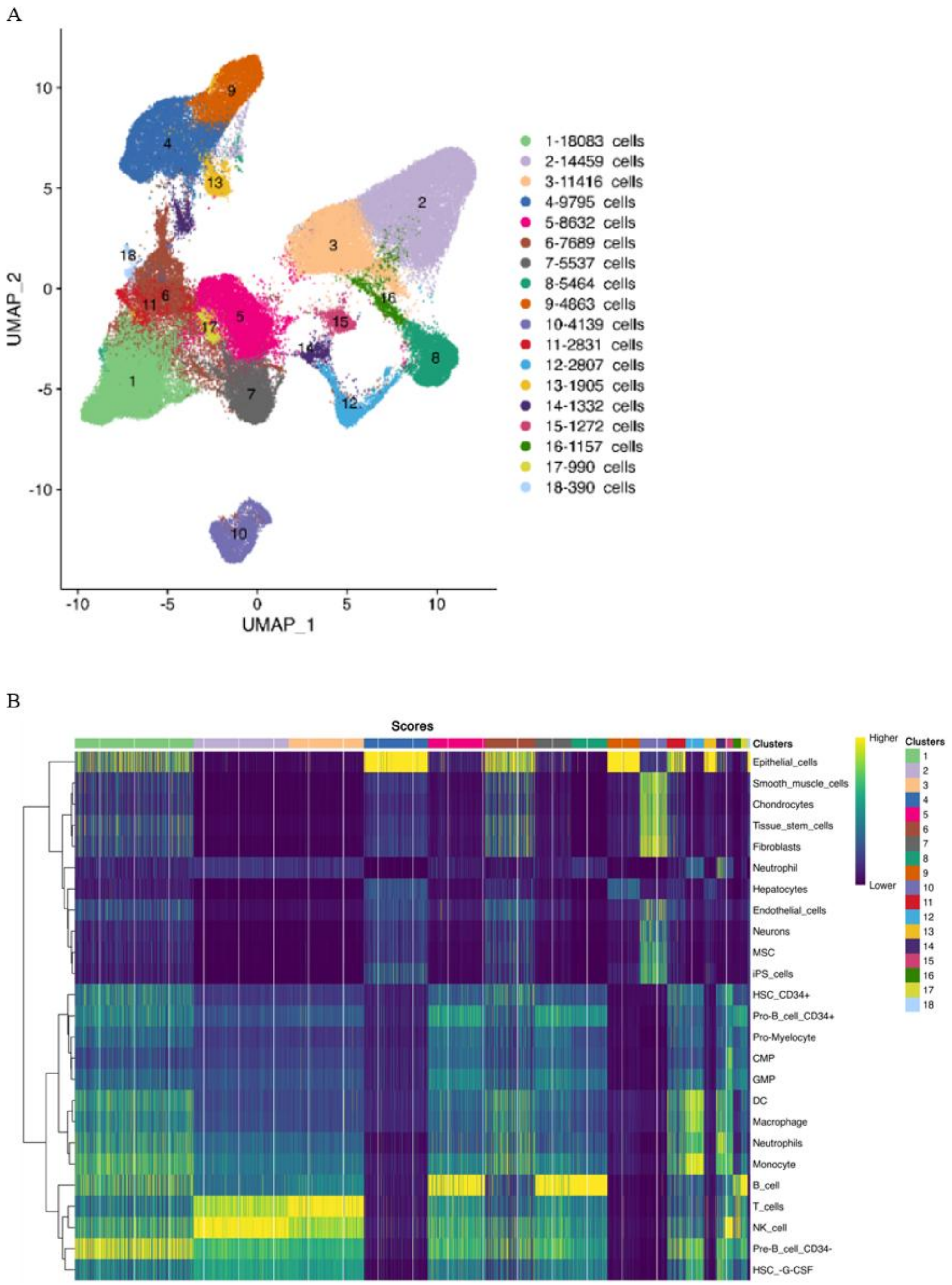

Supplementary Fig. S2. (A) Dimensionality reduction clustering results for all cells from the 15 single-cell samples passing quality control. (B) Heatmap showing cell type identification based on marker genes for each group of cells.

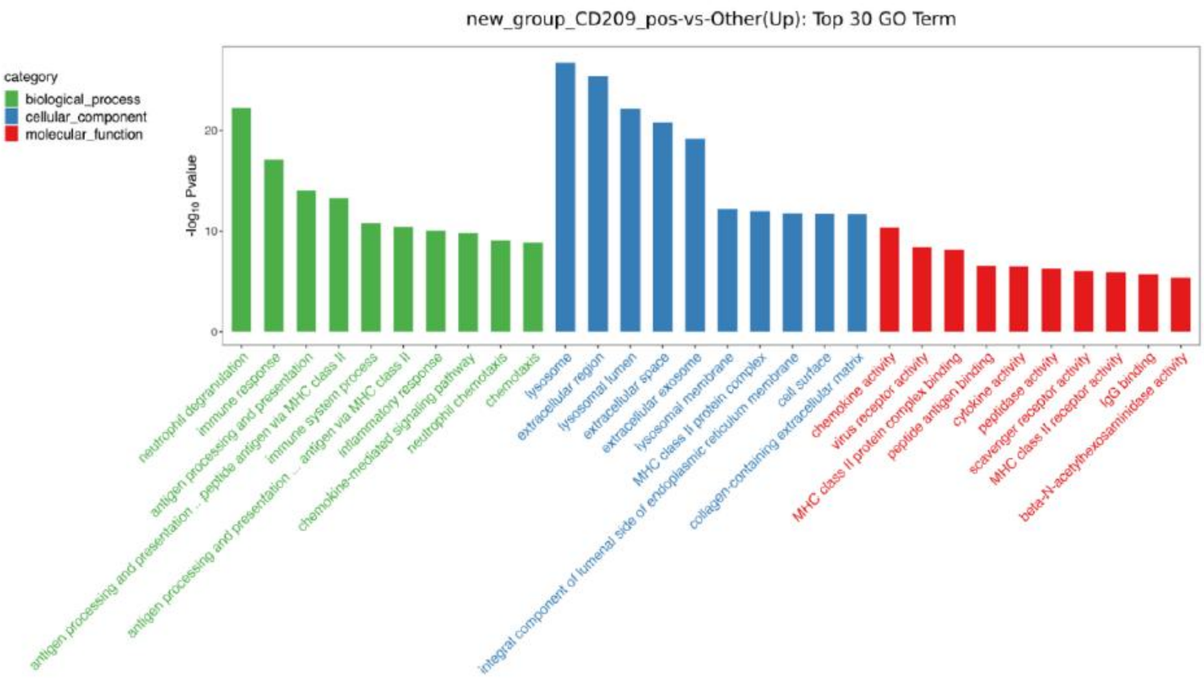

Supplementary Fig. S3. The gene ontology (GO) enrichment analysis of differentially expressed genes between CD209+SIRPα+ double-positive cells and other myeloid cells.

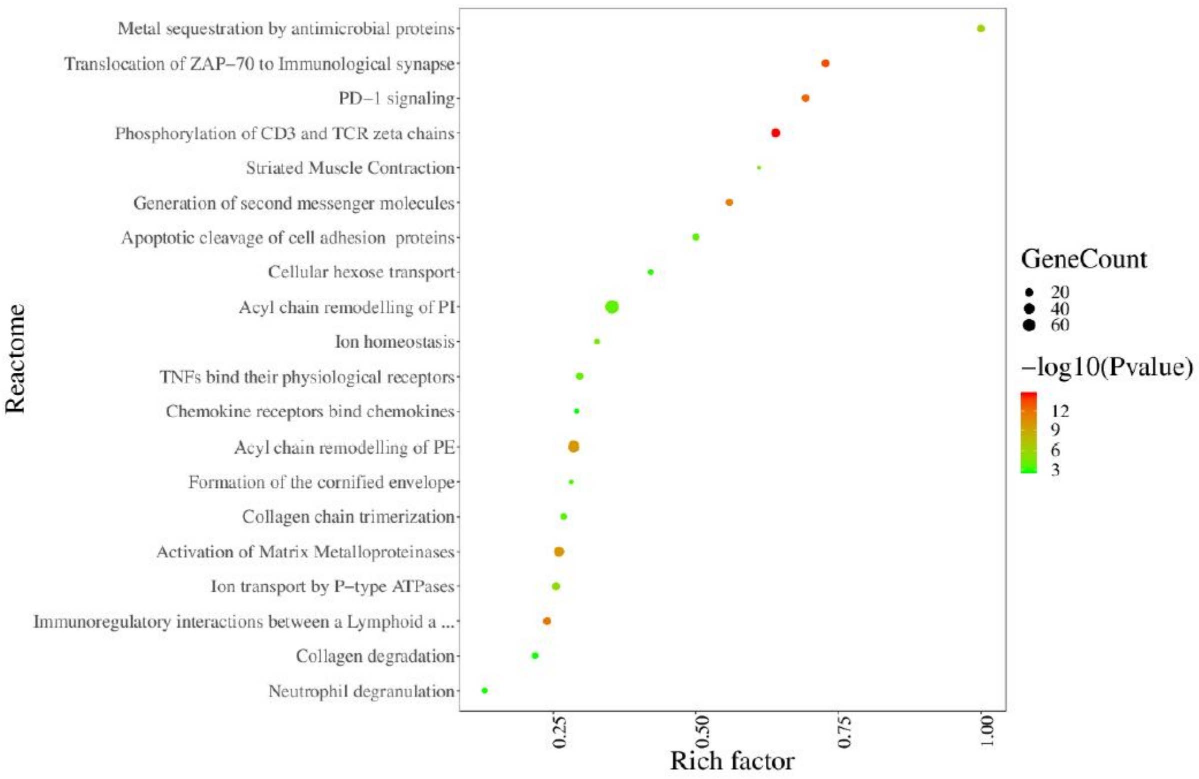

Supplementary Fig. S4. Reactome enrichment analysis comparing differentially expressed genes between the radiation therapy plus SIRPα group and the radiation therapy alone group.
